# Human umbilical cord-derived mesenchymal stem cells ameliorate experimental colitis by normalizing the gut microbiota

**DOI:** 10.1186/s13287-022-03118-1

**Published:** 2022-09-14

**Authors:** Fan Yang, Beibei Ni, Qiuli Liu, Fangping He, Li Li, Xuemei Zhong, Xiaofan Zheng, Jianxi Lu, Xiaoyan Chen, Huizhu Lin, Ruixuan Xu, Yizhan He, Qi Zhang, Xiaoguang Zou, Wenjie Chen

**Affiliations:** 1 Postdoctoral Research Station, Xinjiang Medical University, No. 567 North Shangde Road, Ürümqi, 830018 China; 2 Department of Infectious Diseases, The First People’s Hospital of Kashi, The Affiliated Kashi Hospital of Sun Yat-Sen University, 66 Yingbin Road, Kashi, 844000 China; 3 Biotherapy Centre, The Third Affiliated Hospital, Sun Yat-Sen University, 600# Tianhe Road, Guangzhou, 510630 China; 4 Cell-Gene Therapy Translational Medicine Research Centre, The Third Affiliated Hospital, Sun Yat-Sen University, 600# Tianhe Road, Guangzhou, 510630 China; 5 Department of Respiratory and Critical Care Medicine, The First People’s Hospital of Kashi, The Affiliated Kashi Hospital of Sun Yat-Sen University, 66 Yingbin Road, Kashi, 844000 China; 6 Department of Hepatobiliary and Pancreatic Surgery, The Eighth Affiliated Hospital, Sun Yat-Sen University, Shennan Zhong Road #3025, Futian District, Shenzhen, 518033 Guangdong China

**Keywords:** Mesenchymal stem cells, Crohn's disease, TNBS, 16S rRNA gene sequences, Gut microbiota, Metabolism

## Abstract

**Background:**

Crohn's disease (CD) is a chronic non-specific inflammatory bowel disease. Current CD therapeutics cannot fundamentally change the natural course of CD. Therefore, it is of great significance to find new treatment strategies for CD. Preclinical and clinical studies have shown that mesenchymal stromal cells (MSCs) are a promising therapeutic approach. However, the mechanism by which MSCs alleviate CD and how MSCs affect gut microbes are still unclear and need further elucidation.

**Methods:**

We used 2,4,6-trinitrobenzenesulfonic acid (TNBS) to induce experimental colitis in mice and analysed the microbiota in faecal samples from the control group, the TNBS group and the TNBS + MSC group with faecal 16S rDNA sequencing. Subsequent analyses of alpha and beta diversity were all performed based on the rarified data. PICRUStII analysis was performed on the 16S rRNA gene sequences to infer the gut microbiome functions.

**Results:**

MSC Treatment improved TNBS-induced colitis by increasing survival rates and relieving symptoms. A distinct bacterial signature was found in the TNBS group that differed from the TNBS + MSC group and controls. MSCs prevented gut microbiota dysbiosis, including increasing *α*-diversity and the amount of Bacteroidetes Firmicutes and Tenericutes at the phylum level and decreasing the amount of Proteobacteria at the phylum level. MSCs alleviated the increased activities of sulphur and riboflavin metabolism. Meanwhile some metabolic pathways such as biosynthesis of amino acids lysine biosynthesis sphingolipid metabolism and secondary bile acid biosynthesis were decreased in the TNBS group compared with the control group and the TNBS + MSC group

**Conclusions:**

Overall, our findings preliminarily confirmed that colitis in mice is closely related to microbial and metabolic dysbiosis. MSC treatment could modulate the dysregulated metabolism pathways in mice with colitis, restoring the abnormal microbiota function to that of the normal control group. This study provides insight into specific intestinal microbiota and metabolism pathways linked with MSC treatment, suggesting a new approach to the treatment of CD.

**Supplementary Information:**

The online version contains supplementary material available at 10.1186/s13287-022-03118-1.

## Introduction

Crohn’s disease (CD) is a subtype of inflammatory bowel disease (IBD) characterized by chronic recurrent colonic mucosal inflammation. In CD, inflammation can be found throughout the gastrointestinal tract and it often extends deeper into the colonic tissue layer. IBD has become a global disease with an increasing incidence [1]. Developing countries are in the emergence stage, newly industrialized countries are in the acceleration of incidence stage, and western regions are in the compounding prevalence stage [2]. In western countries, although the incidence is stabilizing, the disease burden remains high, as the prevalence surpasses 0.3% [3].

Treatment for CD includes monoclonal antibody therapies, immunomodulators, and surgery, but the recurrence rate is high, and there is no effective cure [4]. Despite advances in IBD treatment, 30% of patients do not respond to current treatments, and 50% develop allergic reactions or become refractory over time [5]. IBD is a very complex and costly disease, and these data highlight the need for prevention research and health care system innovations.

In the last decade, MSCs have emerged as innovative and promising therapeutic options for many incurable diseases [6–8]. Further interest in MSCs has been attracted by the observation that they exhibit profound effects due to attractive features of plasticity, tropism for inflamed tissues, and high immunomodulatory potential. MSCs are nonhemopoietic cells capable of self-renewal and secreting antibacterial peptides and cytokines [9, 10]. In theory, they can be used as a potential treatment for CD. Clinical studies have suggested the potential benefits of MSC treatment for CD based on their immune regulatory functions [11, 12].

As it shares many features with human CD, the 2,4,6-trinitrobenzenesulfonic acid (TNBS)-induced colitis animal model has been used to study the mechanism and treatment of human CD. Preclinical animal experiments have confirmed that MSCs could significantly improve clinical abnormalities in TNBS-induced colitis mice and reduce lesion scores and inflammation in the gut [13]. However, the mechanism by which MSCs alleviate CD is still unclear and it needs further elucidation.

It is likely that CD is caused by a genetic predisposition combined with environmental triggers that shape the microbiome, such as diet and antibiotic exposure. It is caused by continuous inflammation in response to endogenous microbes in genetically predisposed individuals [14, 15]. The human gut microbiota, composed of trillions of individual microbes, has adapted to the uniquely diverse environments found in the intestine. Gut microbes are an essential part of the microbiota ecosystem. They outnumber human cells by tenfold [16]. In recent years, with the continuous progress of the theoretical research of gut microbiota and the gradual maturity of molecular technology, the relationship between CD and the gut microbiota is slowly being revealed. Jason lloyd-Price et al. [17] provided a comprehensive view of functional dysbiosis in the gut microbiome during IBD activity. The involvement of the gut microbiota in the maintenance of the gut ecosystem is twofold: it educates host immune cells and protects the host from pathogens. However, when the healthy microbial composition and function are disrupted (dysbiosis), the dysbiotic gut microbiota can trigger the initiation and development of various gastrointestinal diseases, including IBD [18].

Experiments on enteric microbiota from IBD patients have revealed that a decreased abundance of the Firmicutes and Bacteroidetes phyla with an increased abundance of Proteobacteria is significantly correlated with the severity of IBD, and genera of the Firmicutes phylum are reduced in IBD patients [19, 20]. Meanwhile, a network of bacteria-metabolite interactions has shown some mechanisms linked to disease activity in Crohn’s disease [21]. Thousands of candidate microbial proteins are likely to interact with the host immune system in IBD [22]. Researchers have found that MSC therapy may be associated with the gut microbiota in many diseases, including ulcerative colitis [23], acute liver injury [24], hypoxia-induced pulmonary hypertension [25], chronic hypoxia [26], and diabetes [27], which leads to gut dysbiosis. Whether the molecular mechanism of MSC therapy for CD is associated with the gut microbiota remains unknown.

Therefore, we hypothesised that treatment of colitis with MSCs could restore the microbiota composition and function in the TNBS-induced colitis model and reduce inflammation to a normal state. Then, we investigated the effects of MSC treatment on host disease status, gut microbiome composition and function using the 16S rRNA gene sequencing method in TNBS-induced colitis to evaluate how the gut microbiome responds to MSC treatment and contributes to colonic inflammatory etiopathogenesis.

## Materials and methods

### Mice

BALB/c mice were purchased from the Model Animal Research Center, Nanjing University (Nanjing, Jiangsu, China). All animals used for the in vivo studies were 8-week-old males. BALB/c mice were randomly allocated to each group. All animal protocols were reviewed and approved by the Sun Yat-sen University Institutional Animal Care and Use Committee.

### Isolation and culture of MSCs

MSCs were isolated and expanded from human umbilical cords according to previously reported protocol [28]. Fresh human umbilical cords were obtained from newborns with parental consent and placed in PBS at 4 °C. The cord was washed twice with PBS to remove any remaining blood. The rinsed cords were cut into 10 mm^3^ pieces and placed in type I collagenase with hyaluronidase (0.1%) containing CaC1_2_ (3 mM) for digestion at 37 °C for 4 h. The specimens were transferred to DMEM (Thermo Fisher Scientific, Waltham, MA, USA) containing 10% foetal bovine serum (Pan Biotech, Aidenbach, Germany) at 37 °C in a humidified atmosphere with 5% CO_2_. The medium in the primary culture was changed 3 days later, and nonadherent cells were removed. After that the culture medium was refreshed every 4 days. Following the appearance of colonies of fibroblast-like cells after 14 days, the cells were trypsinized and transferred to a new flask for further expansion. We incubated the MSCs with 0.05% trypsin–EDTA(GIBCO, Invitrogen Inc., Carlsbad, CA, USA) at 37 °C and stored them at − 80 °C until analysis. For the collection of umbilical cords, the Human Ethics Committee of the Third Affiliated Hospital at Sun Yat-sen University approved this project. Written informed consent was obtained from all participants.

### Flow cytometry

The characteristics of MSCs were identified by flow cytometry and the methods for identifying MSCs were described as previous methods [29]. CytoFLEX flow cytometers (Beckman Coulter) were used for flow cytometry, and CytoExpert software (Beckman Coulter) was used for data analysis. Anti-human CD90-FITC (Catalogue #IM1839U), anti-human CD19-FITC(Catalogue #A07768), anti-human CD11b-FITC(Catalogue #IM0530), anti-human HLA-DR-FITC(Catalogue #IM1638U), anti-human CD34-FITC (Catalogue #IM1870), anti-human CD45-FITC(Catalogue #AO7782), CD105-PE(Catalogue #B76299), and anti-human CD73-PE (Catalogue #B76299) (BECKMAN COULTER, Brea, CA, USA)were purchased from Beckman Coulter.

### Differentiation assays

For osteogenic and adipogenic differentiation procedure of hUC-MSCs was described as previous method [30]. hUC-MSCs were seeded into 24-well plates for osteogenic differentiation and cultured for 12 h at a density of 6 × 10^4^ cells per well. In the following days, the medium was changed to osteogenic differentiation medium (Biological Industries, Kibbutz Beit-Haemek, Israel) and it was refreshed every 3 days. Alizarin red solution (Biological Industries, Kibbutz Beit-Haemek, Israel) was used to stain the induced cells.

To differentiate hUC-MSCs into adipocytes, we seeded them in 24-well plates and cultured them for 12 h at a density of 6 × 10^4^ cells per well. Following that the medium was replaced by adipogenic differentiation medium (Biological Industries, Kibbutz Beit-Haemek, Israel) for 21 days. The medium was then refreshed every 3 days. Oil Red O staining was used on the induced cells.

### Experimental colitis induced by TNBS

For the colitis experiment, the backs of 8-week-old male BALB/c mice were smeared with 150 μl of presensitization solution (Sigma, St. Louis, MO, USA) 7 days before colitis was induced. We divided the mice into groups and fasted them (but still allowed them to drink freely) for 24 h. Our previous study described the procedures for inducing colitis and treating it with MSCs [31]. The parameters of body weight loss, diarrhoea, and survival were recorded daily for 7 days. Three days after TNBS injection (the peak of the disease), the length of the colon was measured from the caecum to the anus. The colons were examined for macroscopic damage based on the protocols described in our previous investigation [31].

### Mesenchymal stem cell transplantation

MSC treatment was performed as described in a previous study [32]. The control mice received only 50% ethanol. MSCs were used for cell transplantation at passages 3–5. After instilling TNBS, the BALB/c mice were either intraperitoneally treated with the control medium (saline) or with 10^6^ MSCs per mouse 2 h later.

### Assessment of colitis severity

The animals were monitored for body weight loss, stool consistency, the presence of blood on the anus or in the stool and survival every day for a total of 7 days. The baseline data were collected before the instillation of TNBS. Disease activity and histologic scores were evaluated as previously described [33]. For disease activity, a scoring system incorporating the percentage of weight loss, stool consistency, and faecal occult blood test results was used [34, 35]. For histopathology analysis, a colon specimen from the middle part (1 cm from the anus to the caecum) was fixed in 10% buffered formalin phosphate and then embedded in paraffin. Sections were stained with haematoxylin and eosin, and inflammation was graded from 0 to 4 as follows, in a blinded fashion: 0, no signs of inflammation; 1, low leukocyte infiltration; 2, moderate leukocyte infiltration; 3, high leukocyte filtration, moderate fibrosis, high vascular density, thickening of the colon wall, moderate goblet cell loss and focal loss of crypts; and 4, transmural infiltrations, massive loss of goblet cells, extensive fibrosis, and diffuse loss of crypts.

### Immunohistochemical staining

Following deparaffinization and rehydration, the tissue sections were placed in a repair box filled with citric acid (pH 6.0) antigen retrieval buffer and heated in a microwave oven to allow antigen retrieval. Then, the slides were transferred into an antigen retrieval solution and incubated with a primary antibody overnight at 4 °C. After incubation with a secondary antibody at room temperature for 1 h, 3,3-diaminobenzidine (Sigma, St Louis, MO) was used to develop the signal. After counterstaining with haematoxylin, optical microscopy was used to examine the sections. The primary antibodies included rabbit anti-human zonula occludens-1 (ZO-1, 1: 150) and rabbit anti-human occludin (1: 100). The antibodies were purchased from Wuhan Servicebio Technology Co., Ltd. The stained sections were read under a microscope and quantified using Image-Pro Plus (IPP) 6.0 software [36]. The quantified results of occludin and ZO-1 expression are presented as the mean density of 5 randomly selected fields from each group.

### Collection and DNA extraction of faecal samples

The faeces of the mice were collected in sterile tubes and stored at – 80 °C until use. Microbial community genomic DNA was extracted from the samples using the E.Z.N.A.® soil DNA Kit(Omega Bio-Tek, Norcross, GA, USA) according to the manufacturer’s instructions. The concentration and purity of the DNA samples were determined using a NanoDrop 2000 (Thermo Fisher Scientific, Wilmington, DE).

### DNA extraction and PCR amplification

The hypervariable region V3-V4 of the bacterial 16S rRNA gene was amplified with the primer pairs 338F (5′-ACTCCTACGGGAGGCAGCAG-3′) and 806R(5′-GGACTACHVGGGTWTCTAAT-3′) by an ABIGeneAmp®9700 PCR thermocycler (ABI, CA, USA). PCR amplification of the16S rRNA gene was performed as follows:initial denaturation at 95 °C for 3 min, followed by 27 cycles of denaturing at 95 °C for 30 s, annealing at 55 °C for 30 s and extension at 72 °C for 45 s, and a single extension at 72 °C for 10 min, ending at 4 °C. The PCR mixtures contained 4 μL of 5 × TransStart FastPfu buffer, 2.5 mM dNTPs 2 μL, 0.8 μL of forward primer (5 μM), 0.8 μL of reverse primer (5 μM), 0.4 μL of TransStart FastPfu DNA Polymerase, 10 ng of template DNA, and ddH2O up to 20 μL. PCRs were performed in triplicate. The PCR product was extracted from a 2% agarose gel, purified using the AxyPrep DNA Gel Extraction Kit (Axygen Biosciences, Union City, CA, USA) according to the manufacturer’s instructions and quantified using a Quantus™ Fluorometer (Promega, Madison, Wisconsin, USA).

### Illumina MiSeq sequencing and processing of sequencing data

The purified amplicons were pooled equimolarly and sequenced on an Illumina MiSeq PE300/NovaSeq PE250 platform (Illumina, San Diego, USA) according to the standard protocols by Majorbio Bio-Pharm Technology Co. Ltd. (Shanghai, China). The raw reads were deposited into the NCBI Sequence Read Archive (SRA) database.

The raw 16S rRNA gene sequencing reads were demultiplexed, quality-filtered by fastp version 0.20.0 [37] and merged by FLASH version 1.2.7 [38] with the following criteria: (1) the 300 bp reads were truncated at any site receiving an average quality score of < 20 over a 50 bp sliding window, and the truncated reads shorter than 50 bp were discarded. Reads containing ambiguous characters were also discarded; (2) only overlapping sequences longer than 10 bp were assembled according to their overlapped sequence. The maximum mismatch ratio of the overlap region was 0.2. Reads that could not be assembled were discarded. (3) Samples were distinguished according to the barcode and primers, and the sequence direction was adjusted, with exact barcode matching and 2 nucleotide mismatches in primer matching.

Operational taxonomic units (OTUs) with a 97% similarity cut-off [39] were clustered using UPARSE version 7.1, and chimeric sequences were identified and removed. The taxonomy of each OTU representative sequence was analysed by Ribosomal Database Project (RDP) Classifier version 2.2 [40] against the 16S rRNA database (e.g., Silva v138) using a confidence threshold of 0.7.

The observed species, Sob, Shannon and Shannoneven are used to evaluate the complexity of species diversity. By performing linear discriminant analysis effect size (LEfSe), we identified the features contributing to the most variation between the control and treatment groups (linear discriminant analysis, LDA > 3). Phylogenetic Investigation of Communities by Reconstruction of Unobserved States (PICRUStII) was further used for genome prediction of microbial communities in this study (Microbiota Sequencing) and then we performed functional categorization according to KEGG Orthology. STAMP3 was used for functional profiling [41].

### Statistical analysis

Data in this study were analysed using IBM SPSS 21.0 software. All results are expressed as the mean ± SD. Tukey's post hoc multiple comparison tests were used to compare three groups, while Student's t tests were used for comparisons between only two groups. Analyses and graphs were generated using GraphPad Prism version 5.01. The relative abundance of bacterial groups at the phylum and genus levels between groups was tested by means of one-way nonparametric Analysis of Variance (ANOVA) (Kruskal–Wallis H test). *P* < 0.05 was considered significant. For the LEfSe test, the nonparametric factorial Kruskal–Wallis (KW) sum-rank test was used to detect significant differences in abundance and to identify the different groups. The Benjamini–Hochberg FDR method was used to correct significant *P* values associated with microbial clades identified by LEfSe. STAMP was used to assess the significance of *P* values associated with microbial function using one-way ANOVA followed by Tukey–Kramer post hoc analyses. The multiple comparisons were not corrected.

## Results

### Identification of hUC-MSC phenotypes

The hUC-MSCs showed a fibroblast-like morphology, expressed certain antigens (CD90, CD105 and CD73, ≥ 95% positive) (Additional file 1: Fig. S1a, g, h), and lacked haematopoietic lineage markers (CD11b, CD34, CD19, HLA-DR and CD45, ≤ 2% positive). The MSCs showed a spindle-shaped and fibroblast-like morphology (Additional file 1: Fig. S1i). After inducing osteogenesis and adipogenesis, mineral accumulation and bone nodule formation were identified by Alizarin red staining (Additional file 1: Fig. S1j), and the hUC-MSCs formed numerous neutral lipid droplets in the cytoplasm, as identified by Oil Red O staining (Additional file 1: Fig. S1k).

### hUC-MSCs alleviate TNBS-induced colitis

**Fig. 1:**
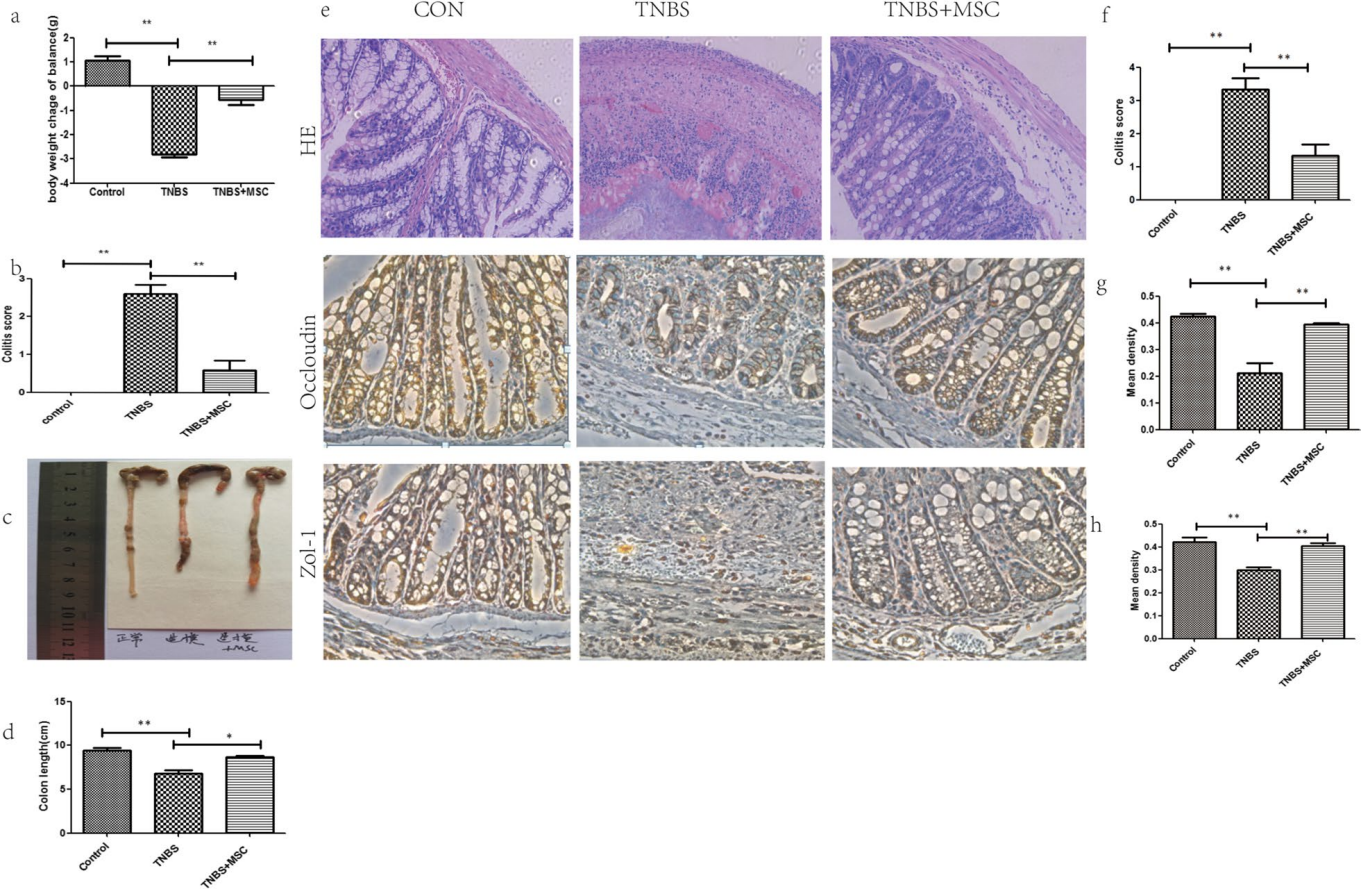
Human umbilical cord-derived mesenchymal stem cell (hUC-MSC) therapy mitigates TNBS-induced colitis. Colitis BALB/c mice were treated with hUC-MSCs (10^6^ per mouse), while the control mice received only control medium (saline) 2 h following rectal administration of 2,4,6-trinitrobenzenesulfonic acid (TNBS). The animals were monitored every day for a total of 7 days for weight loss, the consistency of their stool, the presence of blood in their stool, disease activity and histologic scores, and the expression of mucosal barrier proteins. **a** Decreased weight loss; **b **alleviated colitis symptoms; **c**, **d** Colons were examined for their general form and length 3 days after TNBS intracolonic administration; **e** Histopathologic analysis (H&E staining and histological score). Inflammation was graded from 0 to 4; **f** Expression of the tight junction-related proteins occludin and ZO-1. The stained sections were read under a microscope and quantified using Image-Pro Plus (IPP) 6.0 software. The quantified results of occludin(**g**) and ZO-1(**h**) are presented as the mean density. The mean densities of 5 randomly selected fields in each group. Data are shown as the mean ± SEM. *P < 0.05, **P < 0.01, ***P < 0.001, and n.s. means not significant

Mice treated with TNBS developed severe symptoms, including bloody diarrhoea, rectal prolapse, pancolitis, and sustained weight loss. Similar to the control mice, hUC-MSC treated mice had rapidly recovered body weight loss and milder inflammation. The disease activity index (DAI) score is an indicator of the severity of colitis and is based on results including weight loss, stool features and faecal occult blood. The DAI score for the TNBS group was significantly increased compared with that of the control group, while the DAI scores following treatment with MSC were significantly reduced compared with those of the TNBS group. (Fig. 1b). The colon tissue of the control group was normal in length, uniform in thickness, transparent and smooth, and light yellowish-white. The colon of the model group was thicker and shorter. Numerous ulcers and erosions were observed 2–4 cm from the anus. The colon injury in the hUC-MSC group was significantly reduced and mostly manifested as scattered congestion and erosion (Fig. 1c).

Histopathological staining was used to evaluate the histological characteristics of the colon samples. The results indicated that mice maintained an integrated normal colonic structure in the control group, but mice with TNBS-induced colitis exhibited significant inflammatory cell infiltration, loss of crypts, destruction of the mucosal layer, and oedema (Fig. 1e). In contrast, TNBS-induced colitis in mice treated with hUC-MSCs exhibited mild inflammation. ZO-1 and occludin are important TJ proteins that maintain the integrity of the intestinal mucosal barrier and play an important role in maintaining intestinal permeability. Immunohistochemistry was used to determine the tissue distribution of occludin and ZO-1 in the colon of the mice. Immunohistochemistry showed positive staining for occludin and ZO-1 in the colon of the control group. However, the expression of these two proteins in the colon of the TNBS-induced colitis group was barely detected. TNBS-induced colitis in mice treated with hUC-MSCs resulted in mild occludin and ZO-1 levels (Fig. 1g, h).

### Alpha and beta diversity among the three groups

**Fig. 2:**
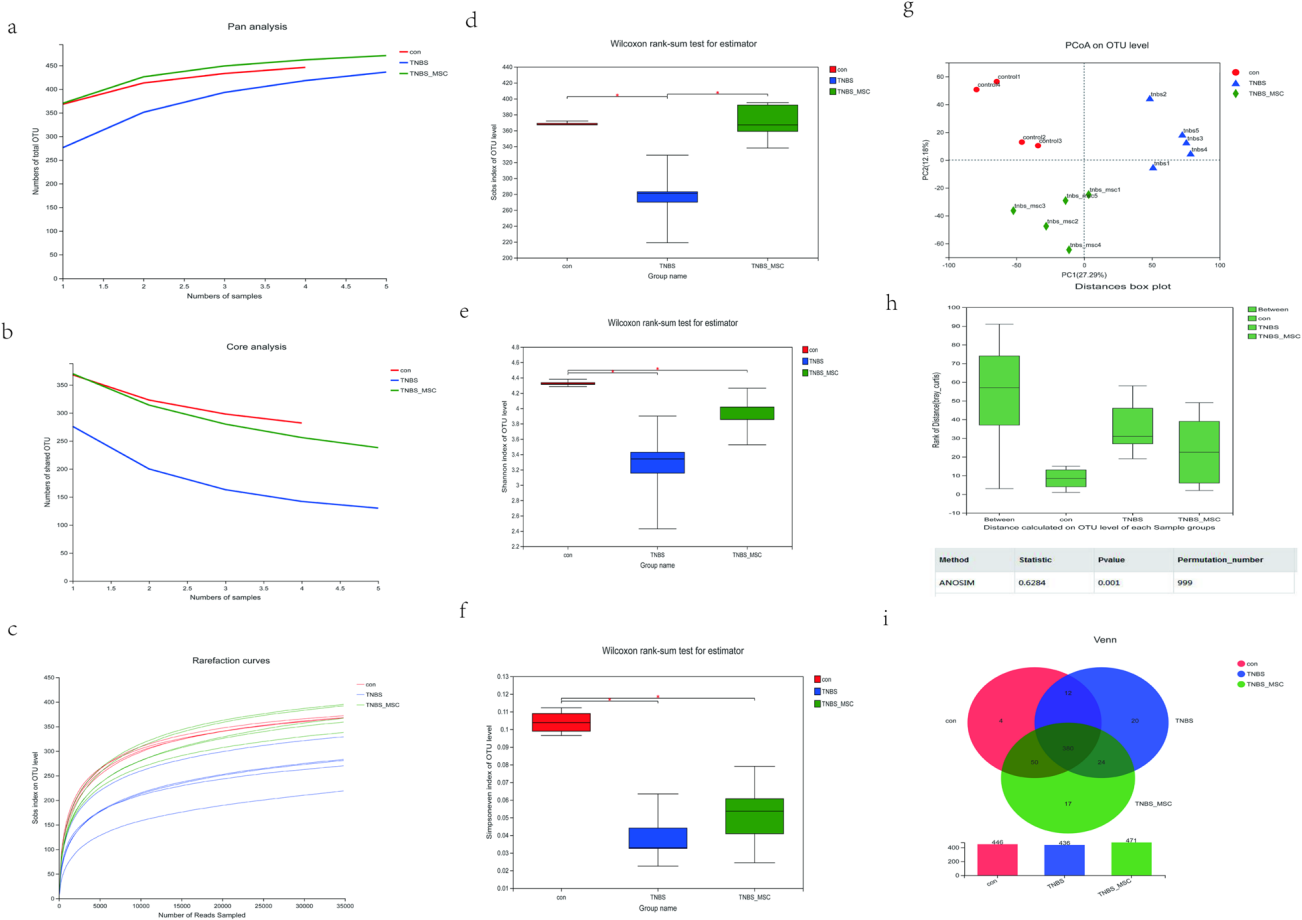
Changes in faecal microbial diversities among the control group (*n* = 4), TNBS group (*n* = 5) and TNBS + MSC group (*n* = 5). Faecal pellets were collected 3 days after MSC injection from the control TNBS and TNBS + MSC groups, and 16SrRNA sequences were determined as described in the "Methods". Alpha-diversity analysis and beta-diversity analysis among the three groups. **a** Pan genome analysis, **b** core genome analysis, **c** rank abundance curve of bacterial OTUs among the three groups. **d**–**f** The Sob, Shannon and Shannoneven indices were used to estimate the diversity of the faecal microbiota among the three groups (data expressed as the mean ± SD). **g** The plots shown were generated using principal coordinate analysis (PCoA). **h **Adonis shows that the difference between groups is significantly greater than that within groups (*P* < 0.001). **i** The Venn plot can be used to count the number of species that are common and unique among the three groups

**Fig. 3:**
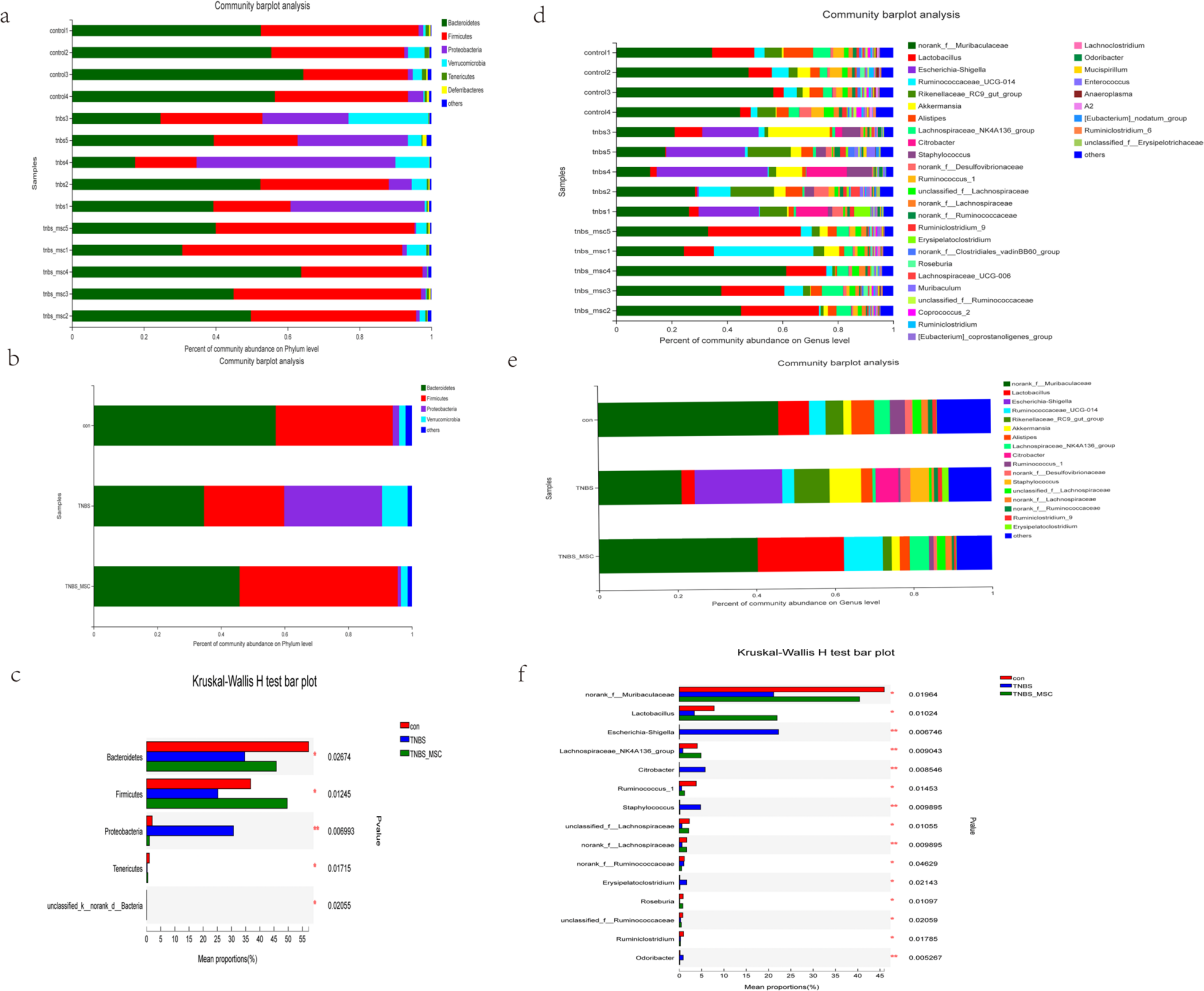
Structural comparison of the faecal microbiota among the control group (*n* = 4), TNBS group (*n* = 5) and TNBS + MSC group (*n* = 5). **a **Clustering of bacterial microbial composition at the phylum level in different samples. **b **The bacterial microbial composition in different experimental groups at the phylum levels. **c **The relative abundance of bacterial groups at the phylum level between groups tested by means of one-way nonparametric Analysis of Variance (ANOVA) (Kruskal–Wallis H test). *P < 0.05, **P < 0.01, ***P < 0.001. **d** Clustering of the bacterial microbial composition at the genus level in different samples. **e** The composition of bacterial microbial composition in different experimental groups at the genus level. **f** The relative abundance of bacterial groups at the genus level between groups tested by one-way nonparametric ANOVA(Kruskal–Wallis *H* test). *P < 0.05, **P < 0.01, ***P < 0.001

Pan/Core analysis was used to annotate the OTU species taxonomy and count the corresponding abundance information of each OTU annotation. When the species curve was flat, it indicated that the numbers of samples were sufficient for sequencing. Therefore, Pan/Core analysis can be used to evaluate whether the number of samples is sufficient for sequencing. Pan/Core analysis showed that the species curve was flat (Fig. 2a, b), indicating that the numbers of samples were sufficient for sequencing. Meanwhile, the rarefaction curve had already reached stable values in the current sequencing. The sequencing depth covered rare new phylotypes (Fig. 3c).

The Sob diversity index indices were significantly lower in the TNBS group than in the control and TNBS + MSC groups (*P* < 0.05 and *P* < 0.05, respectively) (Fig. 2d). Meanwhile, the Shannon and Shannoneven diversity index indices of the TNBS group were significantly lower than those of the control and TNBS + MSC groups (*P* < 0.05 and *P* < 0.05, respectively, Fig. 2e, f). A PCoA plot based on weighted UniFrac distance analysis was used to evaluate the beta diversity. As shown in Fig. 2g, an apparent clustering pattern was identified for the red, blue and green points, which represented the control group, the TNBS group and the TNBS + MSC group, respectively. Analysis of similarities (ANOSIM) suggested that the bacterial microflora composition difference between groups was greater than that within groups (*P* = 0.001) (Fig. 2h). Venn diagrams were constructed to evaluate the number and identity of the shared species among the control group, TNBS group and TNBS + MSC group. Venn diagram analysis revealed that the OTUs were shared by the three groups (Fig. 2i). Together, the results of the alpha and beta diversity analyses revealed significant microbiota variation in the gut microbiome among the control, TNBS and TNBS + MSC groups.

### Gut microbiota differences among the three groups

Phylogenetic analysis of the gut microbiome from the phylum to the species level was performed on the samples using standard operational taxonomic unit (OTU) classification. A distinct bacterial signature was found in the TNBS group that differed from the TNBS + MSC group and controls. The TNBS group was particularly enriched with bacteria in the phylum Proteobacteria. At the phylum level, mice with colitis had decreased abundance of the phyla Bacteroidetes, Firmicutes and Tenericutes compared with control or MSC treated mice (Fig. 3a–c). A similar lineage pattern was seen at the genus level, as Escherichia-Shigella and Citrobacter were enriched in the TNBS group. At the genus level, mice with colitis had decreased abundance of Tenericutes, Lactobacillus, and Ruminococcus (Fig. 3d–f).

**Fig. 4:**
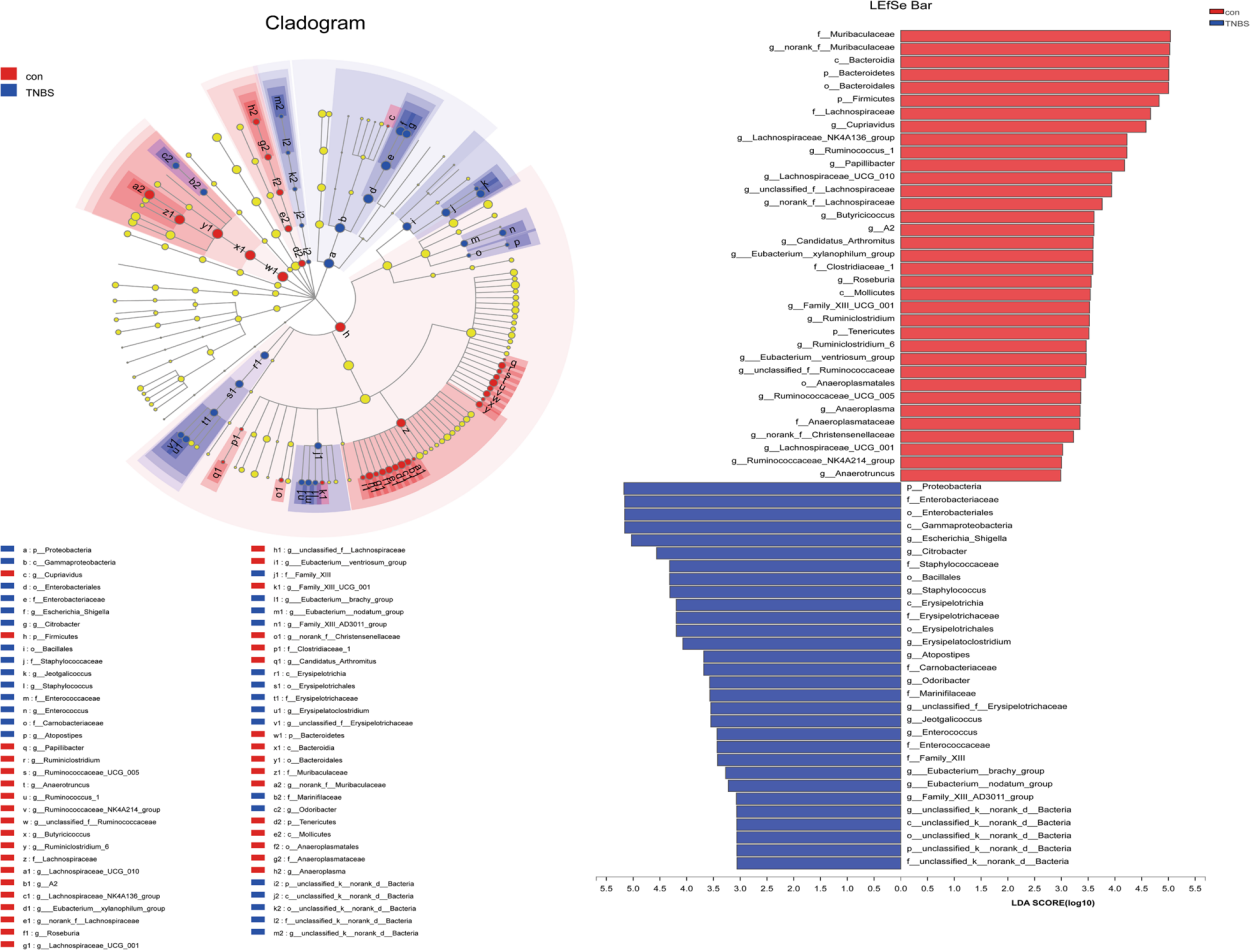
Differences in dominant microorganisms between the control and TNBS groups. In the comparisons of the control group versus the TNBS groups, colitis mice were enriched in the Proteobacteria phylum based on the LDA scores and cladogram. **a** LDA scores from LefSe analysis were performed on relative OTU abundances. Least discriminant analysis (LDA) effect size taxonomic cladogram comparing all samples categorized by control (red) and TNBS groups (blue). **b** Distribution histogram based on LDA (LDA score > 3). Significantly discriminant taxon nodes are coloured, and the branch areas are shaded according to the highest-ranked variety for that taxon. For each taxon detected, the corresponding node in the taxonomic cladogram is coloured according to the highest-ranked group for that taxon. If the taxon is not significantly differentially represented between sample groups, the corresponding node is coloured yellow

We used LEfSe to identify the specific bacterial phylotypes altered by TNBS administration and MSC treatment. To investigate the effects of experimental colitis on the microbiome, the differentiated taxa between the control group and the TNBS group were detected. The cladogram representative of the structure and its dominant bacteria is shown in Fig. 4. In comparisons of the control group versus the TNBS groups, linear discriminant analysis (LDA) scores and the corresponding cladogram showed that colitis mice were most enriched in the Proteobacteria phylum, which included the Gammaproteobacteria class, Enterobacteriaceae order, Enterobacteriales family, Escherichia_Shigella genus, and Citrobacter genus. However, the control group had higher enrichment of the Bacteroidetes phylum, which includes the Bacteroidia class, Bacteroidales order, Muribaculaceae family, and the norank_f_Muribaculaceae genus; and the Tenericutes phylum, which includes the Mollicutes class, Anaeroplasmatales order, Anaeroplasmataceae family, and the Anaerotruncus genus.

**Fig. 5:**
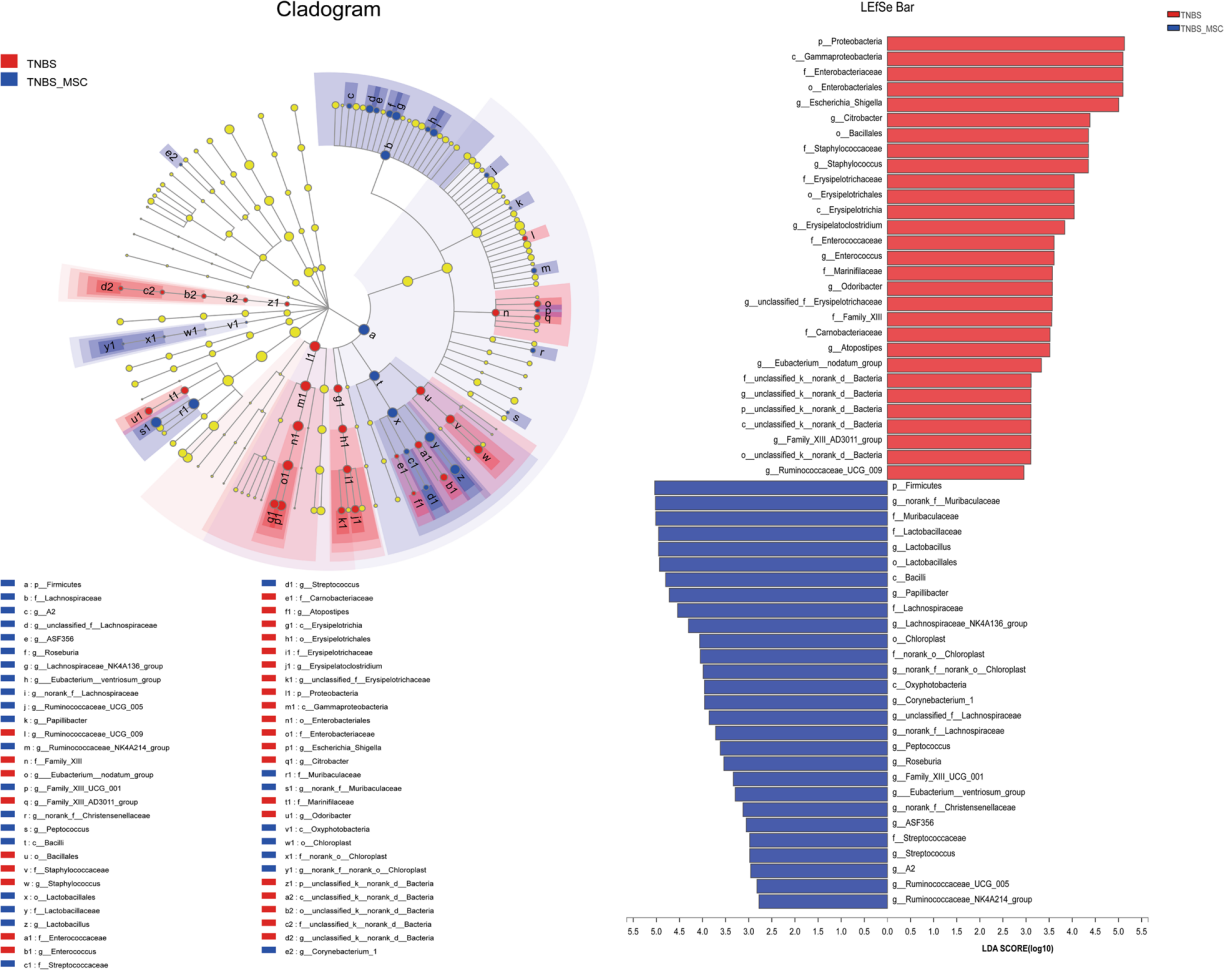
Differences in dominant microorganisms between the TNBS and TNBS + MSCgroups. The TNBS group had higher enrichment of the Proteobacteria phylum, while the TNBS + MSC group had higher enrichment of the Firmicutes phylum **a** LDA scores from LefSe analysis were obtained from the relative OTU abundances and the least discriminant analysis (LDA) effect size taxonomic cladogram comparing all samples categorized by TNBS (red) and the TNBS + MSC group (blue). **b** Distribution histogram based on LDA (LDA score > 3). Significantly discriminant taxon nodes are coloured and the branch areas are shaded according to the highest-ranked variety for that taxon. For each taxon detected, the corresponding node in the taxonomic cladogram is coloured according to the highest-ranked group for that taxon. If the taxon is not significantly differentially represented between the sample groups, the corresponding node is depicted in yellow

Moreover, to investigate the effects of the MSC on the microbiome, the differentiated taxon between the TNBS + MSC group and the TNBS group was assessed (Fig. 5). The TNBS group had higher enrichment of the Proteobacteria phylum, which includes the Gammaproteobacteria class, Enterobacteriaceae order, Enterobacteriales family, Escherichia_Shigella genus, and Citrobacter genus. However, the TNBS + MSC group had a higher enrichment of the Firmicutes phylum, which includes the Bacilli class, Lactobacillales order, Lactobacillaceae family, and Lactobacillus genus. Based on the results among the three groups, it was discovered that MSC treatment could restore the gut microbiome of mice with experimental colitis to a similar composition to the control group.

### Microbial metabolic functions associated with MSC treatment in TNBS-induced colitis

**Fig. 6:**
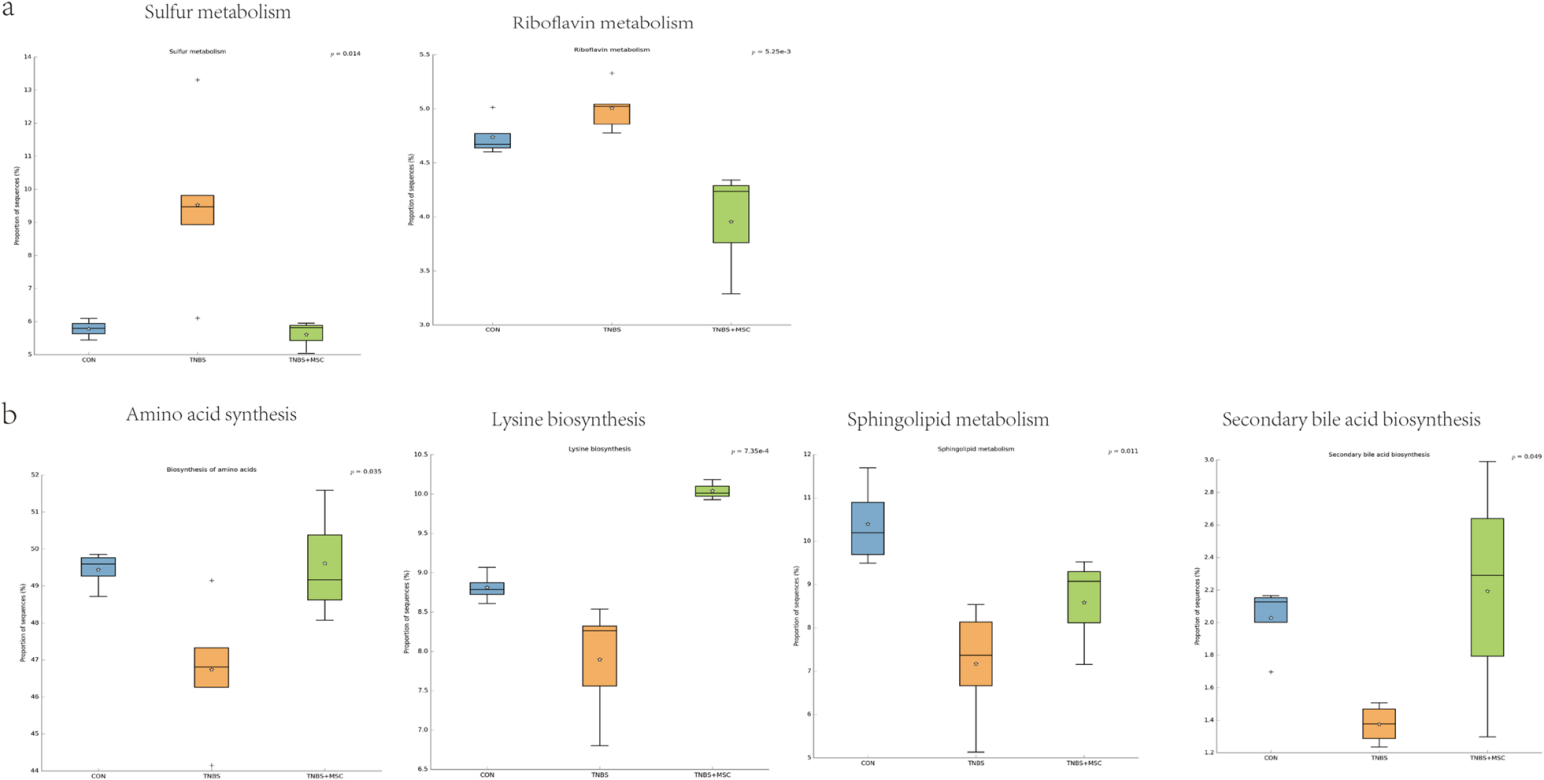
Gut microbiome functions inferred by PICRUStII from 16S rRNA gene sequences among the three groups (the control group, the TNBS group and the TNBS + MSC group). **a** The blue, golden yellow and green bar graphs indicate the inferred metabolism value of control group, TNBS group and TNBS + MSC group. KEGG orthology was used for functional categorization. STAMP3 was used for functional profiling. Elevated metabolic pathways after modelling. **b** Decreased metabolic pathways after modelling. A box plot shows the top quartile, median, and bottom quartile, while white stars indicate the average and a plus sign indicates the outlier. All differences were analysed using one-way ANOVA followed by the Tukey–Kramer post hoc test. The multiple comparisons were not corrected

To investigate the gut microbiome functions related to TNBS administration and MSC treatment, we adopted PICRUStII to infer putative metagenomes from 16S rRNA gene profiles. STAMP was used to identify microbially relevant functions linked with the TNBS administration of MSCs. These pathways were further analysed at the KEGG level. This analysis permitted a comparison of the differences in the functional profiles among all groups and revealed pathways that were significantly different among the control, TNBS and TNBS + MSC groups (Fig. 6). Increased metabolism was present in the TNBS group compared with the control group and the TNBS + MSC group. As a result, MSC decreased the increased activities of sulphur metabolism and riboflavin metabolism (Fig. 6a). The increased levels of riboflavin and sulphur metabolism in the model group may indicate that the biosynthesis of compounds beneficial to oxidative stress has increased. An increase in those compounds may be due to a shift towards an inflammation-promoting microbiome. Some metabolic pathways were decreased in the TNBS group compared with the control group and the TNBS + MSC group, such as the biosynthesis of amino acids, lysine biosynthesis, sphingolipid metabolism, and secondary bile acid biosynthesis (Fig. 6b). The data show that amino acid, lysine, and sphingolipid metabolism, as well as secondary bile acid biosynthesis, were disrupted in the TNBS group. These metabolic changes were reversed by MSCs.

## Discussion

In the present study, we utilized a mouse model of TNBS-induced colitis. Mice were treated with MSCs to determine the beneficial effects of MSCs on the gut microbiota. First, we present evidence that MSCs ameliorate gut dysbiosis and impairment of the intestinal mucosal barrier. Furthermore, PICRUStII analysis data showed that amino acid, lysine, and sphingolipid metabolism, as well as secondary bile acid biosynthesis, were disrupted in the TNBS group. These metabolic changes can be reversed by MSCs.

The human body is colonized by a large number of microbes coexisting peacefully with their host. The most colonized site is the gastrointestinal tract. More than 70% of the microbes in the human body are found in the colon. The microorganism population is 10× larger than the total number of somatic and germ cells in the human body. Two bacterial phyla, making up more than 90% of the microbial cells, dominate the healthy adult intestine: Firmicutes and Bacteroidetes [42]. The microbiota acts as a barrier to pathogens, exerts important metabolic functions, and regulates the inflammatory response by stimulating the immune system [43]. The composition of the gut microbiota exhibits broad interindividual and intraindividual variability [44] and is thought to be a crucial determinant of host susceptibility to several diseases, including IBD. A key feature of IBD is alterations of the composition of the gut microbiota (known as dysbiosis) [45]; however, the precise role of dysbiosis in the disease remains poorly understood. Dysbiosis can destroy these mutualistic relationships and influence the host physiology, compromising human health status [46].

The pathogenesis of IBD in genetically susceptible hosts has been proposed to begin with a breakdown of the intestinal epithelial barrier, followed by a disproportionate immune response to the enteric microbiota, which results in a loss of intestinal homeostasis [47]. Such dysbiosis in the intestinal symbionts of IBD patients can be indicated by the low-level alpha diversity and it manifests as a decrease in many beneficial bacteria, including Firmicutes and Bacteroidetes, and an increased abundance of Proteobacteria [48, 49]. There were also significant changes in both the taxonomic structure and the functional composition of intestinal microbes in TNBS-induced colitis mice [50, 51]. Similar to previous clinical and animal research, our current results showed that TNBS treatment caused microbiota dysbiosis by reducing alpha diversity. At the phylum level, the TNBS group was characterized by enrichment in Proteobacteria and a reduction in Firmicutes and Bacteroidetes. The distribution of bacterial microbiota in TNBS-induced colitis mice was similar to that of CD patients.

Here, we demonstrated that MSC administration significantly ameliorated the loss of body weight and intestinal inflammation and decreased DAI in TNBS-induced colitis mice. After MSC administration, the phyla Bacteroidetes, Firmicutes and Tenericutes were upregulated, and the phylum Proteobacteria was downregulated. Linear discriminant analysis was performed to identify any key microbial biomarkers that might differentiate the disease and MSC treated states. In comparisons of the control versus TNBS groups, LDAs cores and the corresponding cladogram showed that colitis mice were most enriched in the Proteobacteria phylum, which included the Gammaproteobacteria class, Enterobacteriaceae order, Enterobacteriales family, Escherichia_Shigella genus, and Citrobacter genus. However, the control mice had higher enrichment of Muribaculaceae, Bacteroidetes, and Firmicutes. When comparing TNBS with the TNBS + MSC group, Firmicutes had the highest LDA score and enrichment in the MSC-treated mice.

Collectively, these data show that a combination of Proteobacteria phyla, which include the Gammaproteobacteria class, Enterobacteriaceae order, Enterobacteriales family, Escherichia_Shigella genus, and Citrobacter genus, could serve as potential biomarkers of colitis. Effective treatment with MSC was characterized by the enrichment of Firmicutes and Bacteroidetes. MSCs can modulate the gut microbiome during colitis in such a way that they prevent the overgrowth of pathogenic bacteria.

Researchers demonstrated that the microbiota of dextran sodium sulphate (DSS)-induced colitis mice was partially restored after treatment with MSCs [23]. DSS mice have symptoms similar to those of human ulcerative colitis, and TNBS mice have symptoms similar to those of human Crohn’s disease [52]. Our study has implications for understanding whether MSCs can affect gut microbiota in a Crohn's disease mouse model. In accordance with DSS-induced colitis, our study indicates that MSCs also restored the normal microbiota in TNBS-induced colitis mice.

The potential mechanism may be associated with the gut microbiota and host metabolism interaction, as the gut bacteria often target the host metabolism, which further drives immune activation and chronic inflammation [53]. Some pathways and genes were overrepresented and underrepresented in the TNBS group. Based on functional analysis, it is evident that the activities of sulphur and riboflavin metabolism increased while the biosynthesis of amino acids, lysine biosynthesis, sphingolipid metabolism, and secondary bile acid biosynthesis declined.

The TNBS group has an enhanced capacity for managing oxidative stress, a feature of the inflammatory environment, as indicated by incremental riboflavin and sulphur metabolism in the TNBS group compared with the control group and the TNBS + MSC group. Oxidative stress increases in response to inflammation, which allows the microbiota to maintain homeostasis. Thus, the increases in sulphur and riboflavin metabolism may reflect a mechanism by which the gut microbiome addresses the oxidative stress caused by inflammation.

IBD is also associated with a decrease in amino acid synthesis according to previous research [49]. Biosynthesis of amino acids and lysine biosynthesis were reduced in the TNBS group. Under states of inflammation, bacteria typically have a weak ability to produce their own nutrients [54]. The metabolism of amino acids is greatly perturbed. Genes involved in the metabolism and synthesis of nearly all amino acids (particularly lysine) declined in abundance.

Sphingolipid metabolism was decreased in the TNBS group. Sphingolipids play multiple roles in the healthy gut, including membrane structural components in intestinal cells and assigning molecules involved in cell fate decisions [55]. Previous research suggested that sphingolipid metabolism might be disrupted in IBD, causing an accumulation of compounds that promote inflammatory responses [56].

Secondary bile acid biosynthesis declined. Secondary bile acids (SBAs) are produced by bacteria from primary bile acids (PBAs), which have mainly anti-inflammatory effects. It has been found that IBD patients have disrupted BA metabolism, with faecal BA pools skewed towards lower SBAs than healthy controls [57], which was in agreement with our results.

Overall, the functional variation of microbiota among the control group, the TNBS group, and the TNBS + MSC group may indicate that MSC treatment could modulate the metabolism pathways in mice with colitis, restoring the abnormal microbiota function to a normal situation similar to the control group.

These results suggest that perturbations in the bacterial composition are associated with gut microbiome function. Overall, the functional variation of microbiota among the control group, the TNBS group, and the TNBS + MSC group may indicate that MSC treatment could modulate the metabolism pathways in mice with colitis, restoring the abnormal microbiota function to a normal situation as seen in the control group. Limitations in the current mouse experiments are that a mouse model may not fully mimic human Crohn’s disease, and the gut microbiota is different between humans and mice. Clinical studies in CD patients are a future requirement.

## Conclusions

In summary, TNBS treatment induced colonic injury and an inflammatory response in mice. MSCs exerted a protective effect by repairing the intestinal mucosal barrier. MSCs notably restored the alpha diversity and reversed the alterations in the abundance of the gut microbiota in the mice treated with TNBS. MSC treatment could modulate the dysregulated metabolic pathways in mice with colitis. The mechanism might be associated with the gut microbiota, as MSCs improved gut microbiota communities in TNBS-induced murine colitis. To our knowledge, our study is the first to demonstrate MSC treatment and changes in the gut microbiota in a TNBS-induced colitis mouse model. These findings provide insight into specific intestinal microbiotas and metabolic pathways linked with MSC treatment, suggesting a new approach to treating CD. Thus, future perspectives include the following: clinical studies will need to be conducted to evaluate the efficacy and safety of MSCs and determine whether MSCs will normalize the gut microbiota in CD patients.

## Supplementary Information

**Additional file 1**: **Fig. S1** Surface marker expression and characterization of human umbilical cord-derived mesenchymal stromal cells (hUC-MSCs). hUC-MSCs at passage 3 were analysed using flow cytometry, and their osteogenic and adipogenic differentiation was confirmed using Alizarin red staining and Oil Red O, respectively. Flow cytometric analysis of surface markers (**a**. CD90, **b**. CD19, **c**. CD11b, **d**. HLA-DR, **e**. CD34, **f**. CD45, **g**. CD105, **h**. CD73), **i**. fibroblast-like morphology of MSCs, **j**. osteogenic differentiation (Alizarin red staining), **k**. Adipogenic differentiation (Oil Red Ostaining).

## Data Availability

All data and materials generated or used during the study are available from the corresponding authors upon reasonable request.

## Abbreviations

hUC-MSCs: Human umbilical cord-derived mesenchymal stem cells
CD: Crohn’s disease
IBD: Inflammatory bowel disease
TNBS: 2,4,6-Trinitrobenzenesulfonic acid
OUT: Operational taxonomic unit
ZO-1: Zonula occludens-1
LEfSe: Linear discriminant analysis effect size
LDA: Linear discriminant analysis
PICRUSt: Phylogenetic investigation of communities by reconstruction of unobserved states
DAI: Disease activity index
IPP: Image-pro plus
PCoA: Principal coordinates analysis
PBAs: Primary bile acids
SBAs: Secondary bile acids
DSS: Dextran sodium sulphate
RDP: Ribosomal database project
ANOSIM: Analysis of similarities
ANOVA: Analysis of variance
