# Supplementary material for: Human umbilical cord-derived mesenchymal stem cells ameliorate experimental colitis by normalizing the gut microbiota
Source: Stem Cell Res Ther. 2022 Sep 14;13:475. doi: 10.1186/s13287-022-03118-1 (PMC9476645; doi:10.1186/s13287-022-03118-1)
Supplement: Supplementary file 1 — Additional file 1: Fig. S1 Surface marker expression and characterization of human umbilical cord-derived mesenchymal stromal cells (hUC-MSCs). hUC-MSCs at passage 3 were analysed using flow cytometry, and their osteogenic and adipogenic differentiation was confirmed using Alizarin red staining and Oil Red O, respectively. Flow cytometric analysis of surface markers (a. CD90, b. CD19, c. CD11b, d. HLA-DR, e. CD34, f. CD45, g. CD105, h. CD73), i. fibroblast-like morphology of MSCs, j. osteogenic differentiation (Alizarin red staining), k. Adipogenic differentiation (Oil Red Ostaining). [file 13287_2022_3118_MOESM1_ESM.docx]

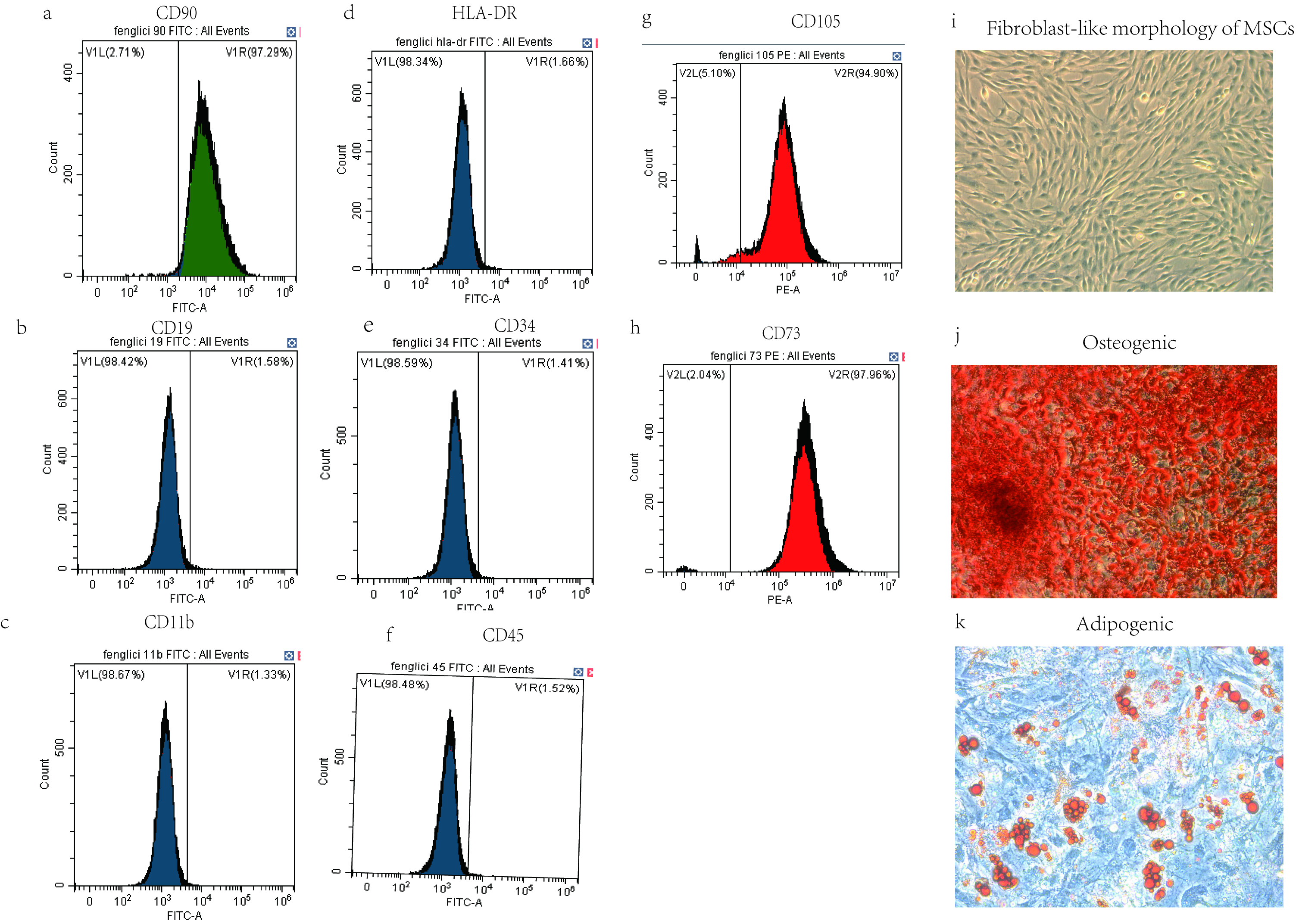


Figure S1 Surface marker expression and characterization of human umbilical cord-derived mesenchymal stromal cells (hUC-MSCs). hUC-MSCs at passage 3 were analysed using flow cytometry, and their osteogenic and adipogenic differentiation was confirmed using Alizarin red staining and Oil Red O, respectively. Flow cytometric analysis of surface

markers(a.CD90,b.CD19,c.CD11b,d.HLA-DR,e.CD34,f.CD45, g.CD105,h.CD73), i.fibroblast-like morphology of MSCs, j.osteogenic differentiation (Alizarin red staining), k.Adipogenic differentiation (Oil Red Ostaining).
